# Comparison of three ^18^F-labeled carboxylic acids with ^18^F-FDG of the differentiation tumor from inflammation in model mice

**DOI:** 10.1186/s12880-016-0110-7

**Published:** 2016-01-12

**Authors:** Hongliang Wang, Ganghua Tang, Kongzhen Hu, Tingting Huang, Xiang Liang, Zhifang Wu, Sijin Li

**Affiliations:** Department of Nuclear Medicine, First Hospital of Shanxi Medical University, Taiyuan, People’s Republic of China; Department of Nuclear Medicine, The First Affiliated Hospital of Sun Yat-Sen University, Guangzhou, People’s Republic of China

**Keywords:** ^18^F-fluoroacetate, 2-^18^F-fluoropropionic acid, 4-^18^F-fluorobenzoic acid, Biodistribution, PET imaging

## Abstract

**Background:**

The aim of this study was to compare the properties and feasibility of the glucose analog, 2-^18^F-fluoro-2-deoxy-D-glucose (^18^F-FDG), three short ^18^F-labeled carboxylic acids, ^18^F-fluoroacetate (^18^F-FAC), 2-^18^F-fluoropropionic acid (^18^F-FPA) and 4-(^18^F)fluorobenzoic acid (^18^F-FBA), for differentiating tumors from inflammation.

**Methods:**

Biodistributions of ^18^F-FAC, ^18^F-FPA and ^18^F-FBA were determined on normal Kunming mice, and positron emission tomography (PET) imaging with these tracers were performed on the separate tumor-bearing mice model and inflammation mice model in comparison with ^18^F-FDG.

**Results:**

Biodistribution results showed that ^18^F-FAC and ^18^F-FPA had similar biodistribution profiles and the slow radioactivity clearance from most tissues excluding the in vivo defluorination of ^18^F-FAC, and ^18^F-FBA demonstrated a lower uptake and fast clearance in most tissues. PET imaging with ^18^F-FDG, ^18^F-FAC and ^18^F-FPA revealed the high uptake in both tumor and inflammatory lesions. The ratios of tumor-to-inflammation were 1.63 ± 0.28 for ^18^F-FDG, 1.20 ± 0.38 for ^18^F-FAC, and 1.41 ± 0.33 for ^18^F-FPA at 60 min postinjection, respectively. While clear tumor images with high contrast between tumor and inflammation lesion were observed in ^18^F-FBA/PET with the highest ratio of tumor-to-inflammation (1.98 ± 0.15).

**Conclusions:**

Our data demonstrated ^18^F-FBA is a promising PET probe to distinguish tumor from inflammation. But the further modification of ^18^F-FBA structure is required to improve its pharmacokinetics.

## Background

Positron emission tomography (PET) has been used for assessing neoplasms for several decades. 2-^18^F-fluoro-2-deoxy-D-glucose (^18^F-FDG) is the most widely used PET probe to detect cancer based on elevated glucose metabolism in the malignant tissue (Warburg effect), as a result of increased expression of cellular membrane glucose transporters (mainly transporter 1) and enhanced hexokinase II enzymatic activity in tumor cells [1, 2]. There are some limitations of ^18^F-FDG in tumor imaging, such as the uptake in activated inflammatory cells causing the false-positive results [3, 4], the low specificity for justified detection of some tumors [5], and the high accumulation in the brain limiting the detection of brain metastases [6, 7]. Given the limitations of ^18^F-FDG PET imaging, the novel imaging agents with greater tumor specificity are required.

Some radiolabeled short carboxylic acids have been investigated for tumor imaging. Radiolabeled acetate (such as ^11^C-acetate) has been used for many years as a probe for measuring myocardial oxidative metabolism and tumor imaging [8–10], but the short half-life of ^11^C (20.4 min) limited its widespread applications. ^18^F-fluoroacetate (^18^F-FAC) has been developed as a mimic of ^11^C-acetate for imaging prostate cancer with higher tumor-to-background ratios than ^11^C-acetate [11]. 2-^18^F-fluoropropionic acid (^18^F-FPA) was another important mimic of ^11^C-acetate, which could delineate both androgen-dependent and androgen-independent prostate xenografts with high tumor-to-background ratios [12]. There was less report about ^18^F-FAC and ^18^F-FPA in inflammation imaging [13]. 4-^18^F-fluorobenzoic acid (^18^F-FBA) was initially developed as a fluorine-18-labeled reagent for coupling with small peptides from solid phase synthesis [14]. To the best of our knowledge, no studies about ^18^F-FBA as a probe in PET imaging have been reported. Therefore, the main purpose of this study was to investigate the biodistribution and PET imaging of these three ^18^F-labeled short carboxylic acids (^18^F-FAC, ^18^F-FPA and ^18^F-FBA) and to evaluate their feasibility for differentiating between tumor and inflammation in model mice in comparison with ^18^F-FDG.

## Methods

### Radiopharmaceuticals

Automated synthesis of ^18^F-FDG was performed on an automatic synthesizer (IBA, Belgium). ^18^F-FAC was produced by radiofluorination of benzyl bromoacetate and followed by deprotection using on-column basic hydrolysis according to the method described by Tang et al. [15]. ^18^F-FPA was prepared according to our previously reported method including nucleophilic fluorination of the precursor methyl-2-bromopropionate and deprotection using on-column basic hydrolysis protocols [16]. ^18^F-FBA was produced via two-step one-pot procedure consisting of radiofluorination of ethyl-4-(trimethylammoniumtriflate) benzoate and basic hydrolysis described by Marik and Sutcliffe [17].

### Animal models

The animal experiments were approved by the Committee on Animal and Human Research at the First Affiliated Hospital, Sun Yat-Sen University. And a completed ARRIVE guidelines checklist for animal experiments is included in Supplementary Material. Female Kunming mice (20–25 g body weight) were obtained from Laboratory Animal Center of Sun Yat-Sen University. Tumor-bearing mice model and inflammation mice model were prepared in different mice as two separate models. S-180 fibrosarcoma cells were cultured in α-minimum essential medium (MEM, containing 10 % fetal bovine serum, 100 U/mL penicillin and 100 μg/mL streptomycin) at 37 °C in a humidified atmosphere with 5 % CO_2_. Thirty mice were subcutaneously injected with S-180 fibrosarcoma cells (6 × 10^6^ cells per mouse) into the right shoulder blade. The mass of the tumors grew to 8–10 mm within two weeks for the experiments.

Sterile inflammation was induced by injecting turpentine oil, which would give rise to chronic inflammation with fibroblasts, vascular proliferation and macrophage infiltration [18]. Turpentine oil (0.2 mL per mouse) were intramuscularly injected into the right thigh muscle of mice (*n* = 30). Initial assessment of lesions was determined by visual examination and palpation, and lesions were allowed to grow for 3 days for the experiments.

### Biodistributions of ^18^F-FAC, ^18^F-FPA and ^18^F-FBA in normal mice

For the biodistribution study, the healthy Kunming mice were intravenously injected with 3.7 MBq of ^18^F-FAC, ^18^F-FPA and ^18^F-FBA. Following the injection, animals were sacrificed at 5 min, 30 min, 60 min and 90 min (*n* = 4 each point). Blood was collected from retro-orbital bleeding, and the other normal tissues of interest, including the heart, brain, liver, kidney, pancreas, intestine, stomach, lung, muscle and bone in normal mice, were rapidly dissected and weighed. The radioactivity was counted with an auto-gamma counter. All measurements were background-subtracted and decay-corrected to the time of injection, then averaged together. Tissue radioactivity was expressed as percentage of injected dose per gram of tissue (% ID/g).

### PET imaging with ^18^F-FAC, ^18^F-FPA, ^18^F-FBA and ^18^F-FDG

PET images were obtained using an Advanced Scanner (GEMINI GXL-16, PHILIPS, Netherlands) in three-dimensional acquisition mode. The experimental animals were deeply anesthetized by intraperitoneal injection of 5 % chloral hydrate (10 mL/kg) before imaging and remained anesthetized through the study. PET scanning was performed on the 3 days after turpentine oil injection and 10 days after tumor implantation. Ten-minute static PET/CT scans were performed at 30 min, 60 min, 90 min and 120 min after intravenous (i.v.) administration of 3.7 MBq of ^18^F-labeled radiopharmaceuticals described above. PET images were reconstructed from the Line of Response (LOR) RAMLA algorithm with low-dose CT images for attenuation correction and localization of the lesion site, resulting in three-dimensional images consisting of 128 × 128 × 90 voxels of 2.0 × 2.0 × 2.0 mm^3^. The regions of interest were placed on each tumor, inflammatory lesion and the region of left thigh muscle. After PET imaging, the animals were sacrificed, some interested tissues (blood, tumors, inflammation region of the right thigh muscle and normal muscle) were excised and weighted, and the radioactivities of these tissues were measured with the gamma counter. The selectivity index (SI) of radiotracers was the tumor-to-inflammation ratio corrected for the background activity of blood or muscle according to the following formula:

$$ \mathrm{S}\mathrm{I}=\frac{\left[\mathrm{Tumor}\;\mathrm{uptake}\hbox{-} \mathrm{Blood}\;\mathrm{or}\;\mathrm{Muscle}\;\mathrm{uptake}\right]}{\left[\mathrm{Inflammation}\;\mathrm{uptake}\hbox{-} \mathrm{Blood}\;\mathrm{or}\;\mathrm{Muscle}\;\mathrm{uptake}\right]} $$

### Histopathology

The excised tumors, inflammation region of the thigh muscle and normal thigh muscle were fixed in formalin and embedded in paraffin. Five micron sections of each tissue were stained with hematoxylin and eosin, and examined for tumor and inflammatory region.

### Statistical analysis

All reported values were expressed as mean ± standard error of the mean. Differences among the various groups (^18^F-FDG, ^18^F-FAC, ^18^F-FPA and ^18^F-FBA tissues uptake after PET imaging) were tested for statistical significance using the Student’s *t* test (or Wilcoxon signed rank test when the data dose not follow a normal distribution.), respectively. And the differences were considered significant at the 95 % confidence level (*p* < 0.05).

## Results

### Radiosynthesis

Starting from benzyl bromoacetate and methyl-2-bromopropionate, the automated synthesis of ^18^F-FAC and ^18^F-FPA were performed in approximately 36 min. And the uncorrected radiochemical yields were 55 ± 5 % and 46 ± 7 % (*n* = 5) at end of synthesis for ^18^F-FAC and ^18^F-FPA, respectively. Using one-pot procedure and purification by SEP-PAK cartridges, the radiochemical yield of ^18^F-FBA was 32 ± 3 % at end of synthesis (*n* = 5, decay uncorrected) within 50 min. The specific activity of ^18^F-FAC, ^18^F-FPA and ^18^F-FBA were >37 GBq/μmol with over than 95 % radiochemical purity.

### Biodistributions of ^18^F-FAC, ^18^F-FPA and ^18^F-FBA in normal mice

**Fig. 1:**
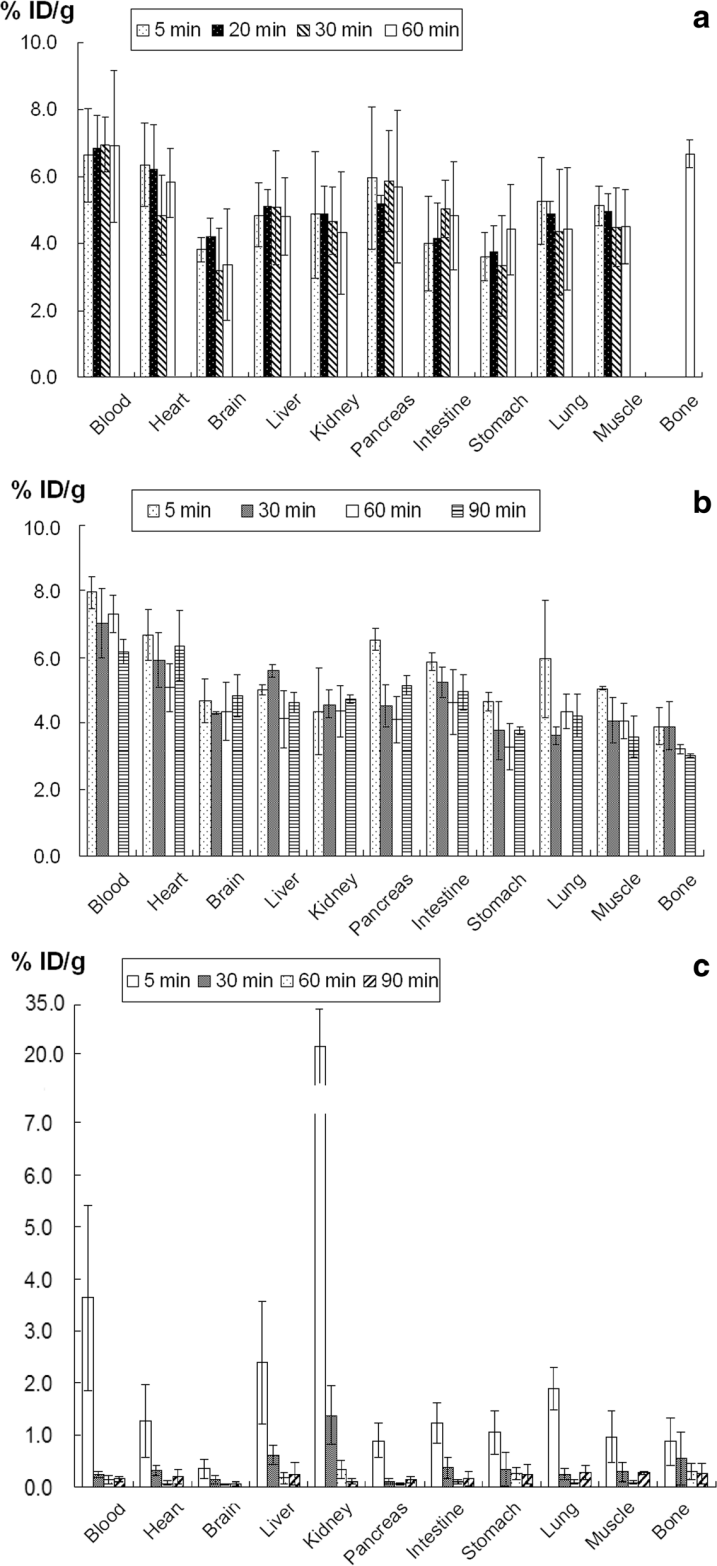
The biodistributions of ^18^F-FAC (**a**), ^18^F-FPA (**b**) and ^18^F-FBA (**c**) in normal Kunming mice

Biodistributions of ^18^F-FAC, ^18^F-FPA and ^18^F-FBA at different time phases in normal mice are shown in Fig. 1. For ^18^F-FAC (Fig. 1a), the elimination of radioactivity from blood was relatively slow within 60 min (6.64 ± 1.40 % ID/g at 5 min vs. 6.92 ± 2.27 % ID/g at 60 min postinjection, *p* > 0.05). In addition, the radioactivity accumulation in liver, lung, heart, muscle and pancreas showed a similar profile of radioactivity clearance as that in blood within 60 min. But a higher radioactivity uptake in bone was found at 60 min postinjection (6.68 ± 0.43 %ID/g). For ^18^F-FPA (Fig. 1b), the uptake of this tracer in most investigated organs remained constant throughout the experiment time of the study, and the distribution profile of ^18^F-FPA in most tissues was similar to that of ^18^F-FAC. However, the lower uptake of ^18^F-FPA in bone was observed with the constant radioactivity accumulation over the whole time of study (3.91 ± 0.57 % ID/g at 5 min vs. 3.02 ± 0.07 %ID/g at 90 min, *p* > 0.05). For ^18^F-FBA, the most tissue uptakes at 60 min postinjection were lower than those of ^18^F-FAC and ^18^F-FPA (Fig. 1c), and the radioactivity clearance of ^18^F-FBA from most tissues was very fast within the experiment time, especially for blood and kidneys (Blood: 3.63 ± 1.77 % ID/g at 5 min vs. 0.14 ± 0.08 % ID/g at 60 min, *p* < 0.05; Kidneys: 21.69 ± 11.3 % ID/g at 5 min vs. 0.34 ± 0.18 % ID/g at 60 min, *p* < 0.05).

### PET imaging with ^18^F-FDG, ^18^F-FAC, ^18^F-FPA and ^18^F-FBA in model mice

**Fig. 2:**
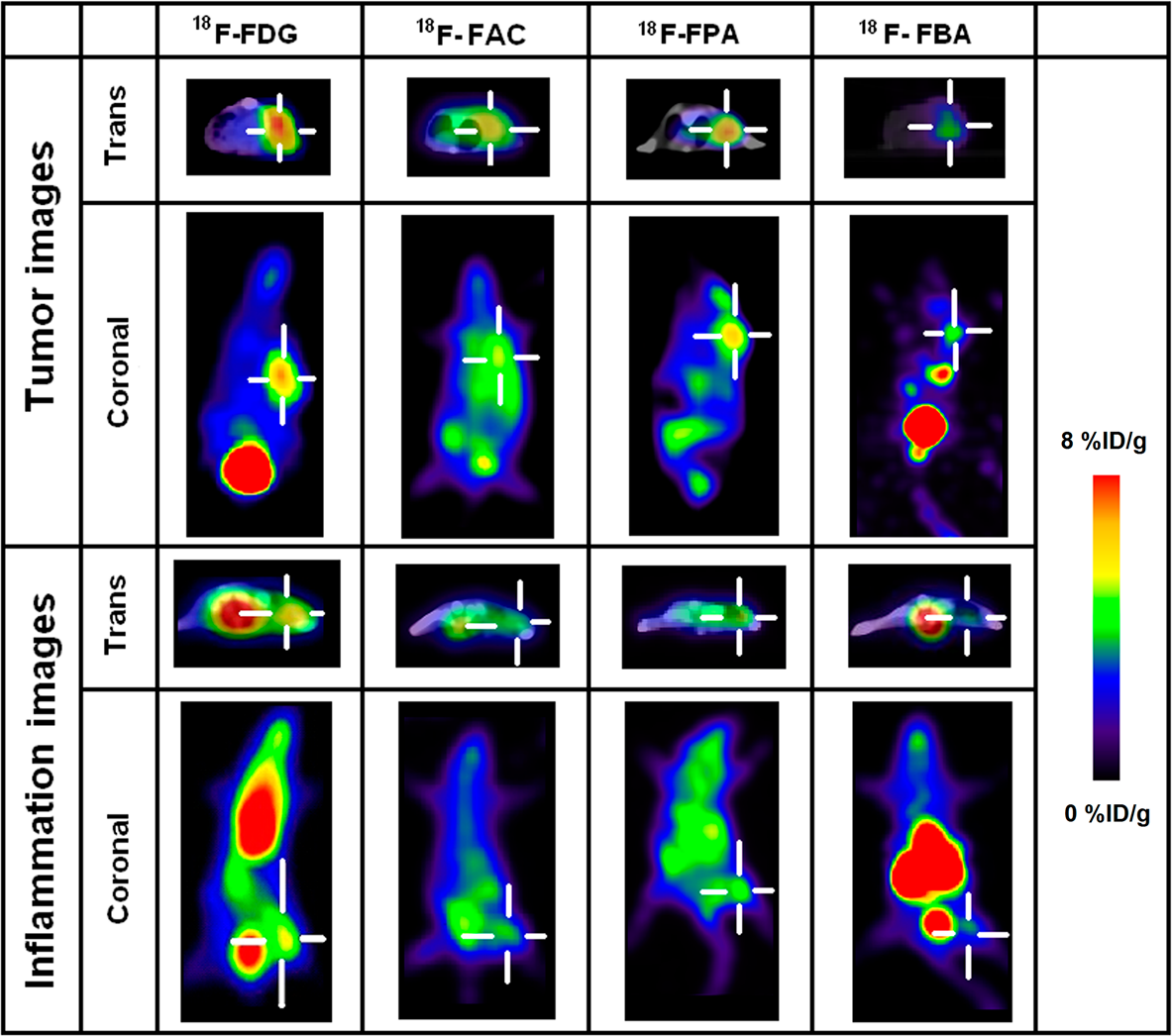
PET images of S180 fibrosarcoma-bearing mice model (*the up two rows*) and sterile inflammation mice model (*the below two rows*) with ^18^F-FDG, ^18^F-FAC, ^18^F-FPA and ^18^F-FBA at 60 min postinjection. Four short white lines indicate the location of tumor or inflammatory lesion areas, respectively

PET images of ^18^F-FDG, ^18^F-FAC, ^18^F-FPA and ^18^F-FBA obtained in tumor-bearing mice and inflammation mice are shown in Fig. 2. In the PET-CT images of tumor-bearing mice, the radioactivity of ^18^F-FDG, ^18^F-FAC and ^18^F-FPA is clearly localized in S-180 tumor on the right shoulder blade. In the PET-CT images of inflammatory mice, the highest uptake of ^18^F-FDG in the inflammatory tissue and the moderate uptake of ^18^F-FAC and ^18^F-FPA were observed. For ^18^F-FBA, the uptake in tumor revealed that ^18^F-FBA accumulation was significantly lower than those of ^18^F-FAC and ^18^F-FPA. However, there is no significant ^18^F-FBA uptake in inflammation lesions.

After PET imaging, the accumulations of radioactivity in blood, brain, liver, muscle, tumor and inflammatory lesions derived from the excised tissues, which were acquired at 60 min postinjection of tracers, are shown in Table 1.

**Table 1:** Accumulation comparison of ^18^F-FAC, ^18^F-FPA, ^18^F-FBA and ^18^F-FDG (3.7 MBq in saline) in model mice (*n* = 3)

	^18^F-FDG	^18^F-FAC	^18^F-FPA	^18^F-FBA
Blood^a^	0.72 ± 0.18	3.36 ± 1.66	4.35 ± 1.09	0.05 ± 0.01
Muscle^a^	1.06 ± 0.47	3.73 ± 0.53	3.87 ± 0.42	0.22 ± 0.02
Tumor^a^	6.67 ± 1.13	6.12 ± 1.76	6.51 ± 1.28	0.63 ± 0.10
Inflammation^a^	4.11 ± 1.96	5.10 ± 0.33	4.62 ± 0.33	0.31 ± 0.04
Tumor-to-Blood^b^	1.68 ± 0.26	1.82 ± 0.45	1.50 ± 0.36	12.60 ± 2.12
Inflammation-to-Blood	1.03 ± 0.14	1.52 ± 0.21	1.06 ± 0.26	6.20 ± 1.06
Tumor-to-Muscle	6.30 ± 0.69	1.70 ± 0.16	1.80 ± 0.19	2.76 ± 0.25
Inflammation-to-Muscle	3.87 ± 0.61	1.42 ± 0.13	1.38 ± 0.13	1.40 ± 0.13
Tumor-to-Inflammation	1.63 ± 0.28	1.20 ± 0.38	1.41 ± 0.33	1.98 ± 0.15*
SI (Blood)^c^	1.76 ± 0.23	1.59 ± 0.12	8.00 ± 1.37*	2.23 ± 0.34
SI (Muscle)^d^	1.84 ± 0.17	1.74 ± 0.16	3.52 ± 0.13*	3.72 ± 0.06*

^a^Values were presented as % ID/g (mean ± SD)

^b^Defined as (Tumor uptake)/(Blood uptake)

^c^Defined as (Tumor uptake − Blood uptake)/ (Inflammation uptake − Blood uptake), that is, Tu/Inf ratio corrected for blood background activity

^d^Tu/Inf ratio corrected for Muscle background activity

*p < 0.05, vs. the ratios for ^18^F-FDG

The comparative orders of tumor-to-muscle (Tu/Mu) ratios were ^18^F-FDG (6.30 ± 0.69) > ^18^F-FBA (2.76 ± 0.25) > ^18^F-FPA (1.80 ± 0.19) > ^18^F-FAC (1.70 ± 0.16). And the highest inflammation-to-muscle (Inf/Mu) ratio was 3.87 ± 0.61 for ^18^F-FDG, and other ratios of Inf/Mu basically were close for ^18^F-FAC (1.42 ± 0.13), ^18^F-FBA (1.40 ± 0.13) and ^18^F-FPA (1.38 ± 0.13). For ^18^F-FAC, the high radioactivity accumulation in muscle made it difficult to differentiate between tumor and inflammation (Tu/Mu vs. Inf/Mu: 1.70 ± 0.16 vs. 1.42 ± 0.13, p > 0.05). Although the ^18^F-FPA had the similar tumor-to-inflammation ratio to that of ^18^F-FAC, the selectivity indices (SI) (Tu/Inf ratios corrected with muscle and blood uptake) of ^18^F-FPA (3.52 ± 0.13 and 8.00 ± 1.37) were significantly higher than those of ^18^F-FAC (1.74 ± 0.16 and 1.59 ± 0.12). For ^18^F-FBA, significant lower radioactivity accumulations were observed in tumor and inflammatory lesion, but the Tumor-to-inflammation (Tu/Inf) ratio of ^18^F-FBA was the highest (1.98 ± 0.15) among these experimental PET tracers due to the lower inflammation uptake. Being corrected with the background uptake of muscle, ^18^F-FBA has a supreme selectivity index (SI) (Muscle corrected, 3.72 ± 0.06) due to its comparatively low radioactivity accumulation in the inflammatory lesion. Our results indicated that ^18^F-FBA was specifically retained in S180 fibrosarcoma lesions.

### Histopathologic findings of tumors and inflammation

**Fig. 3:**
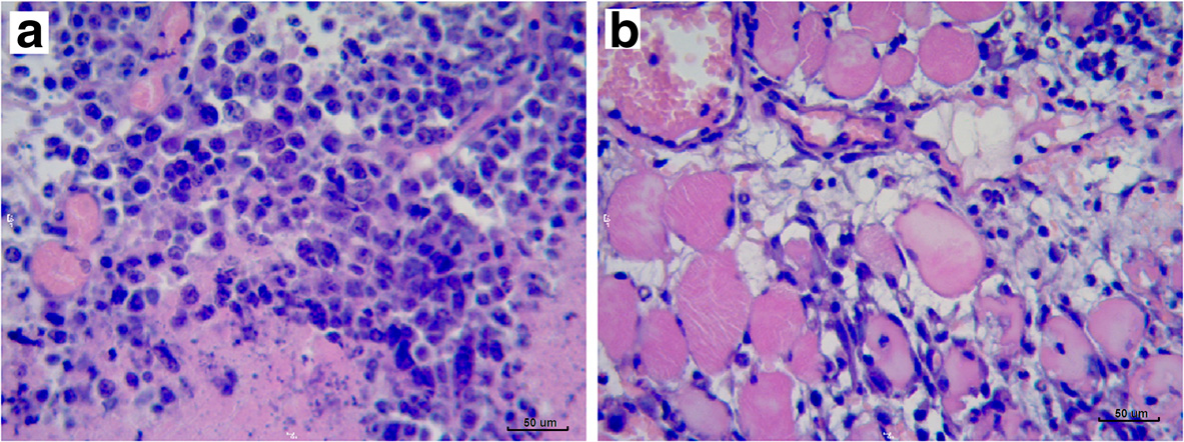
Microscopic histological examination of a specimen excised from tumor and inflammation in mice. A specimen of mice tumor (**a**), 10 days after inoculation of S-180 cells and the mice inflammatory muscle (**b**), 72 h after injection of turpentine oil (all specimens were stained by hematoxylin and eosin and magnified by × 400)

Histological examination revealed malignant mesenchymal tumor in a specimen of the excised S-180 tumors. S-180 tumor cells with pleomorphic, hyperchromatic nuclei were arranged in tumor mass and many mitoses were found (Fig. 3a). As shown in Fig. 3b, histological examination demonstrated inflammatory reaction in a specimen of the turpentine oil-injected muscle, and massive infiltration of neutrophils was seen in and between muscle fibers.

## Discussion

In the view of the positive accumulation of ^18^F-FDG in both tumor and inflammatory cells, the false-positive findings are always accompanied with uptake of ^18^F-FDG in the inflammatory lesions, such as the abscess or granulation tissues surrounding the abscess. Hence, many radiotracers have been developed to use in differentiating tumor from inflammatory lesions [19, 20]. Radiolabeled short carboxylic acids would be an additional tool in characterizing the tumor by measuring the lipid metabolism. Tumor uptake of radiolabeled acetate not only reflects the expression of cytosolic acetyl-coenzyme A (CoA) synthetase [8], but also the fatty acid synthase [21]. The fatty acid synthase is overexpressed in various cancers and could catalyze the de novo synthesis of fatty acids from acetyl-CoA [22, 23]. In this study, the feasibility of differentiation between tumor and inflammation in vivo with positron emitter-labeled short chain alkyl and aryl carboxylic acids was investigated and compared with ^18^F-FDG.

In the biodistribution studies, the two short alkyl carboxylic acids (^18^F-FAC and ^18^F-FPA) showed the similar characteristic of pharmacokinetics. The most tissues performed both higher radioactivity accumulation and slower clearance for ^18^F-FAC and ^18^F-FPA than those of ^18^F-FBA. The main reason for the slow clearance of ^18^F-FAC from those tissues could be attributed to the lack of oxidative metabolism in tissues and the slow transformation of ^18^F-FAC to 2-^18^F-fluoroacetyl-CoA [24], as well as the fluorocitric acid (in vivo) conversed from 2-^18^F-fluoroacetyl-CoA could inhibit the aconitase causing the inhibition of the tricarboxylic acid cycle [24, 25]. Several studies have reported the utilization of propionate as a favoured energy substrate by heart and tumor cells [25–29]. And the metabolism of propionic acid begins with its conversion to propionyl-CoA catalyzed by mitochondrial propionyl-CoA synthetases [30, 31], as the first common step in the metabolism of fatty acids. Therefore, ^18^F-FPA could have a similar metabolic mechanism to ^18^F-FAC. Unlike the significant defluorination of ^18^F-FAC (Fig. 1a), ^18^F-FPA within 90 min postinjection appeared to show no evidence of defluorination (Fig. 1b), which was consistent with previous studies [12]. In contrast, ^18^F-FBA showed a faster radioactivity clearance from most tissues within 60 min postinjection. Meanwhile, most tissues had a lower radioactivity uptake of ^18^F-FBA after 5 min postinjection than those of ^18^F-FAC and ^18^F-FPA, so that ^18^F-FBA performed the low bioavailability and the primarily excretion from kidney, which would result in a massively radioactivity accumulation in bladder (Fig. 2).

In this study, the S-180 fibrosarcoma is a malignant mesenchymal tumor derived from fibrous connective tissue and characterized by the presence of immature proliferating fibroblasts or undifferentiated anaplastic spindle cells in a storiform pattern. And the animal model of inflammation in the current study is chronic inflammation characterized by fibroblast proliferation and neovascularization with mononuclear cell infiltration (macrophages, lymphocytes and plasma cells) [18]. Therefore, we investigated the fibroblasts cells uptake for different probes in these models both with fibroblast proliferation. Our previous reports had shown that ^18^F-FDG/PET exhibited high accumulations both in S-180 fibrosarcoma tumor and inflammation lesions [31, 32]. The ^18^F-FDG uptake in tumor and inflammatory were 6.30-fold and 3.87-fold higher than that in healthy muscle, respectively (Table 1). All these results proved that ^18^F-FDG could not effectively achieve the differentiation between tumor and inflammation [33]. Both ^18^F-FAC and ^18^F-FPA also successfully delineated tumor xenografts and inflammation lesions in model mice. For ^18^F-FPA, the ratio of tumor-to-inflammation was lower than that of ^18^F-FDG (Table 1), but the higher selectivity indices of tumor-to-inflammation (muscle or blood corrected ratios of tumor-to-inflammation, 3.52 ± 0.13 or 8.00 ± 1.37) were observed. The significant uptake of ^18^F-FAC and ^18^F-FPA in the inflammatory lesions might be attributed to the high expression of the acetyl- or propionyl-CoA synthetases and fatty acid synthnase in these tissues [34–36]. In addition, the higher uptake of ^18^F-FAC and ^18^F-FPA were observed in the lower part of the gut, which was a result of the re-absorption of small carboxylic acids occurrence [37].

For ^18^F-FBA, the biodistribution studies demonstrated that ^18^F-FBA had an advantage of rapid clearance in the most tissues. The differences of biodistribution between ^18^F-FBA and ^18^F-FAC (or ^18^F-FPA) could be attributed to the different metabolic pathway, and ^18^F-FBA was converted into *p*-^18^F-fluorohippuric acid derivatives via a glycine-conjugation pathway in the liver and eliminated through the kidneys [38–40]. Meanwhile, ^18^F-FBA PET imaging showed the clear contrast between tumor and inflammation lesion with the highest ratios of tumor-to-inflammation as 1.98 ± 0.15. Although both the fast clearance and low uptake in the most tissues rendered the high selectivity index of ^18^F-FBA (Muscle or Blood corrected ratios of tumor-to-inflammation, 3.72 ± 0.06 or 2.23 ± 0.34), the low radioactivity accumulation in tumor and inflammation would be adverse for the delimitation of the interested tissues in PET imaging. Due to the low uptake of ^18^F-FBA in tumor, some new strategies should be attempted to modify the profiles of ^18^F-FBA to improve its pharmacokinetics. Recently, it was reported that the new benzoic acid derivatives could be conjugated with amino acids to provide new potential PET tracers for tumor detection [41]. However, all these works needed to be further studied.

## Conclusion

In this study, the ex vivo biodistribution and in vivo PET imaging results demonstrate that ^18^F-FBA seems to be a potential PET tracer to detect tumor and to differentiate tumor from inflammation in comparison with ^18^F-FDG and ^18^F-FAC (or ^18^F-FPA). And the structure modification of ^18^F-FBA would be performed to improve the profiles of biodistribution in the further studies.

## Abbreviations

^18^F-FAC: ^18^F-fluoroacetate
^18^F-FBA: 4-(^18^F)fluorobenzoic acid
^18^F-FDG: 2-^18^F-fluoro-2-deoxy-D-glucose
^18^F-FPA: 2-^18^F-fluoropropionic acid
CoA: coenzyme A
PET: positron emission tomography
